# Supplementary material for: Genetic Variations Associated with Drug Resistance Markers in Asymptomatic Plasmodium falciparum Infections in Myanmar
Source: Genes (Basel). 2019 Sep 9;10(9):692. doi: 10.3390/genes10090692 (PMC6770986; doi:10.3390/genes10090692)
Supplement: Supplementary file 1 [file genes-10-00692-s001.pdf]

Table S1. Polymerase chain reaction (PCR) primer sequences for the amplification of sequences containing *P. falciparum* Kelch protein 13 *(pfk13)*, chloroquine resistance transporter *(pfcrt)* and multidrug resistance protein 1 (*pfmdr1*) genes.

Target gene	Amplication	PCR	Primer name	Primer sequences 5'-3'	Product (bp)	Reference
pfk13	K13 Whole gene	Primary	K13_c.-155F	AACAAGGCGTAAATATTCGTGT	2438	[1]
			K13_c.2283R	TGTGCATGAAAATAAATATTAAAGAAG		
	K13 Fragment a	Nested	K13_c.-155F	AACAAGGCGTAAATATTCGTGT	874	
			K13_c.719R	TCTCGAATAAAATTCATTTGTGTCTT		
	K13 Fragment b	Nested	K13_c.614F	TTGAAACGGAATTAAGTGATGC	851	
			K13_c.1464R	CAATACAGCACTTCCAAAATAAGC		
	K13 Fragment c	Nested	K13_c.1344F	AGGTGGATTTGATGGTGTAGAA	940	
			K13_c.2283R	TGTGCATGAAAATAAATATTAAAGAAG		
pfcrt	crt 1-2exons	Primary	E1/2-F	CGACATTCCGATATATTATATTTTTAGAC	740	[2]
			E1/2-R	TATATGTGTAATGTTTTATATTGG		
		Nested	E1/2-NF	CCGTTAATAATAAATACACGCAG	694	
			E1/2-NR	AATGTTTTATATTGGTAGGTGG		
	crt 3-8exons	Primary	E3/8-F	CCACCTACCAATATAAAACATTAC	1446	
			E3/8-R	GTTAAAATATATATAAATGTCTC		
		Nested	E3/8-NF	TATATATATATGTATGTATGTTG	1370	
			E3/8-NR	AATGTCTCTTATAATTTTGAAATT		
	crt 9-13exons	Primary	E9/13-F	CTTATAATAAAATTTCAAAATTATAAGAGAC	1286	
			E9/13-R	GAGATCTCTATACCTTCAACATTATTCC		
		Nested	E9/13-NF	GAGACATTTATATATATTTTAAC	1234	
			E9/13-NR	CCTTATAAACTCTAATGCG		
pfmdr1	mdr1 86-184	Primary A	86-184F	TGTTGAAAGATGGGTAAAGAGCAGAAAGAG	780	[3]
			86-184R	TACTTTCTTATTACATATGACACCACAAACA		
		Nested	184F	AAAGATGGTAACCTCAGTATCAAAGAAGAG	560	
			184R	GTCAAACGTGCATTTTTTATTAATGACCAAAT		
	mdr1 1304-1246	Primary B	1034-1042-1246F	AGAAGATTATTTCTGTAATTTGATAGAAAAAGC	880	
			1034-1042-1246R	ATGATTCGATAAATTCATCTATAGCAGCAA		
		Nested	1042F	TATGTCAAGCGGAGTTTTTGC	337	
			1042R	TCTGAATCTCCTTTTAAGGAC		
		Nested	1246F	GTGGAAAATCAACTTTTATGA	499	
			1246R	TTAGGTTCTCTTAATAATGCT		

Table S2. PCR primer sequences for the amplification of sequences containing *P. falciparum* dihydrofolate reductase *(pfdhfr),* dihydropteroate synthase *(pfdhps)* and multidrug resistance-associated protein 1 (*pfmrp1*) genes.

Target gene	Amplication	PCR	Primer name	Primer sequences 5'-3'	Product (bp)	Reference
pfdhfr	dhfr	Primary	Pfdhfr_D1	TTTATATTTTCTCCTTTTTA	718	[4]
			Pfdhfr_D2	CATTTTATTATTCGTTTTCT		
		Nested	Pfdhfr_M1	TTTATGATGGAACAAGTCTGC	648	
			Pfdhfr_M5	AGTATATACATCGCTAACAGA		
pfdhps	dhps	Primary	Pfdhps_N185	TGATACCCGAATATAAGCATAATG	1151	[4]
			Pfdhps_N218	ATAATAGCTGTAGGAAGCAATTG		
		Nested	Pfdhps_Rc	GGTATTTTTGTTGAACCTAAACG	728	
			Pfdhps_Rd	ATCCAATTGTGTGATTTGTCCAC		
pfmrp1	Fragment a	Primary	1first fw	TGTGTTAATTTGTGATTCCA	1228	[5]
			1first rev	AATACAAGAAGTAACAAAAAATGT		
		Nested	1nest fw	TGTGTTAATTTGTGATTCCA	1205	
			1nest rev	TTACAGACGGTACATCAGAA		
	Fragment b	Primary	2first fw	AAGGTCAAAAGATTCCTGTA	1200	
			2first rev	TTCAAATTATGTGATGTACCA		
		Nested	2nest fw	GATTTCAAAGTATTCAGTGGG	1145	
			2nest rev	ACCATCATCATGAGAAGTTATATTT		
	Fragment c	Primary	3first fw	GTTTTTATAAGTTCAATTTCCTCT	1031	
			3first rev	TCCATGTTCTATGGTACTCAA		
		Nested	3nest fw	ATATAGTTGAAGTGGTTATATTCTTT	927	
			3nest rev	TTTCCAAATAAAATCATTGATCT		
	Fragment d	Primary	4first fw	ATAATAAATGTGATAATGACCATA	1282	
			4first rev	ATAGGTATCAAGCGTATATTCA		
		Nested	4nest fw	AAAAGGAATTCATTAGCAATA	1201	
			4nest rev	GAAGAAACAGTTGGACTATCAT		
	Fragment e	Primary	5first fw	TTTGAAATTCAGGATAAAACTTT	1237	
			5first rev	TGACATGATAAATACAGCCTT		
		Nested	5nest fw	ACTAAAGAAAGTCACTGGGGTTA	1148	
			5nest rev	CAGCCTTTGTGCTTCTTTAC		
	Fragment f	Primary	6first fw	CTTTTTAACTATGATGAGTATTATTTCT	1292	
			6first rev	TCTCTTCCTTCTACTGTTATTTT		
		Nested	6nest fw	AATAAAGAACATTCAGACACAAT	1175	
			6nest rev	TGATTTTCCTACTATCCCAATT		
	Fragment g	Primary	7first fw	CGTTTTCTGCTAGACTAGGTGTTA	1185	
			7first rev	ATTATGTATGCATGGGTGTGTG		
		Nested	7nest fw	AAGAAATGTGCTGTGTTCAAAG	1113	
			7nest rev	GCATGGGTGTGTGTAAGTTT		

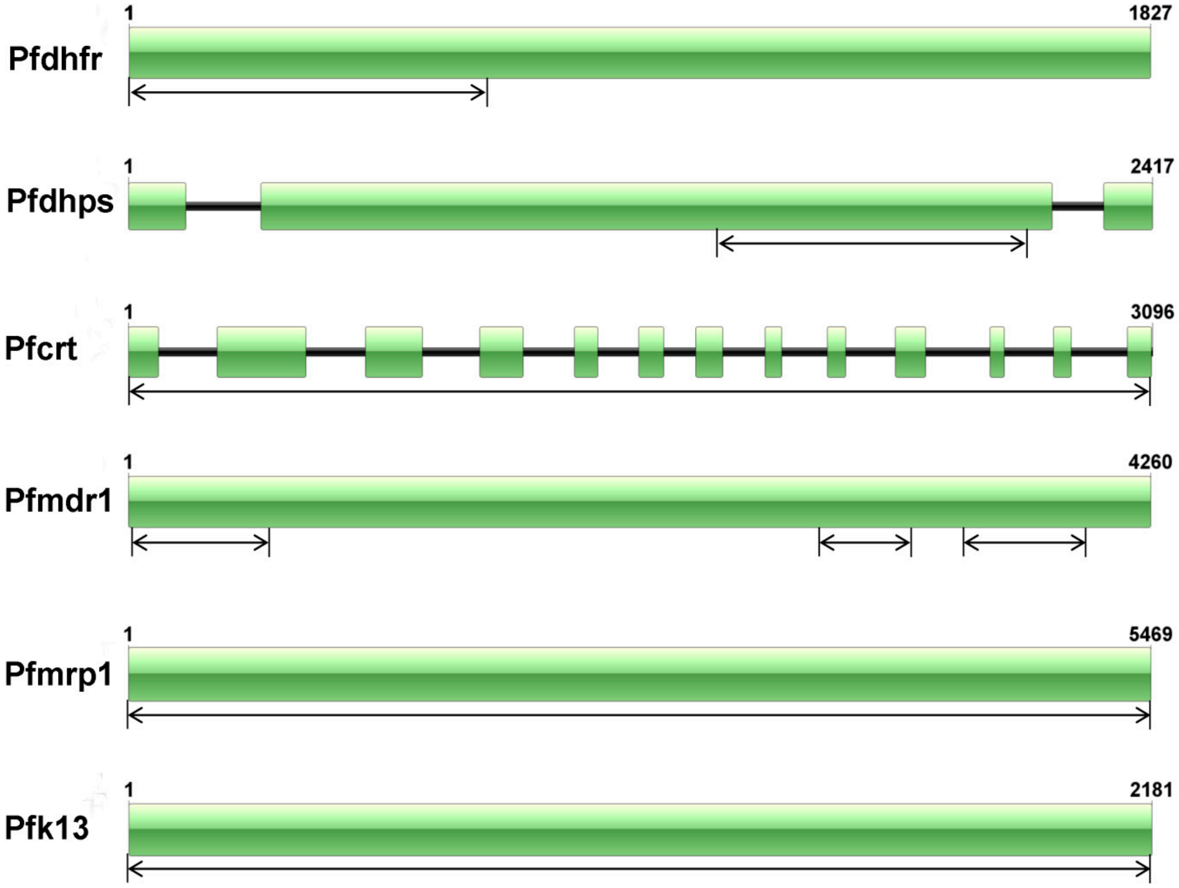

Figure S1. Schematic diagram showing successfully sequenced regions from different genes. Boxes indicate exons, and lines indicate introns. Regions marked by double arrows were amplified successfully.

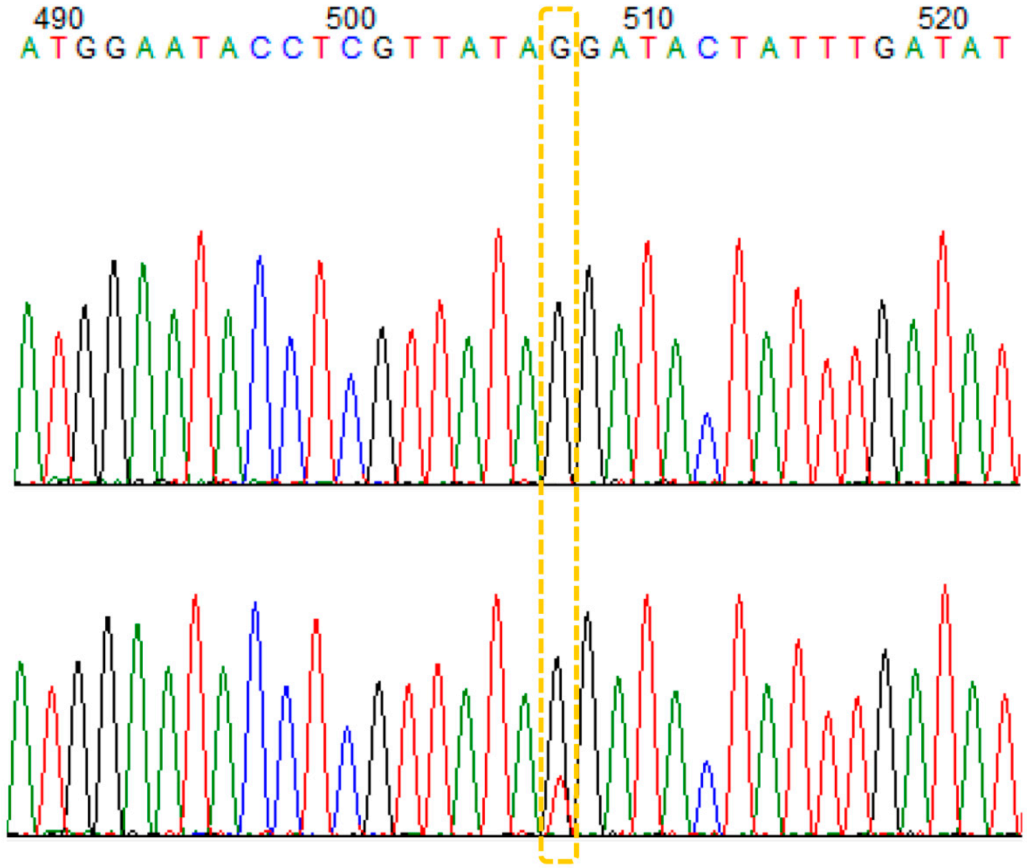

Figure S2. Sequencing chromatograms showing single and mixed infections. The base position indicated by the yellow dotted line shows the sample with single peak indicating monoclonal infection (Top), while the double peaks indicate mixed infection (Bottom).

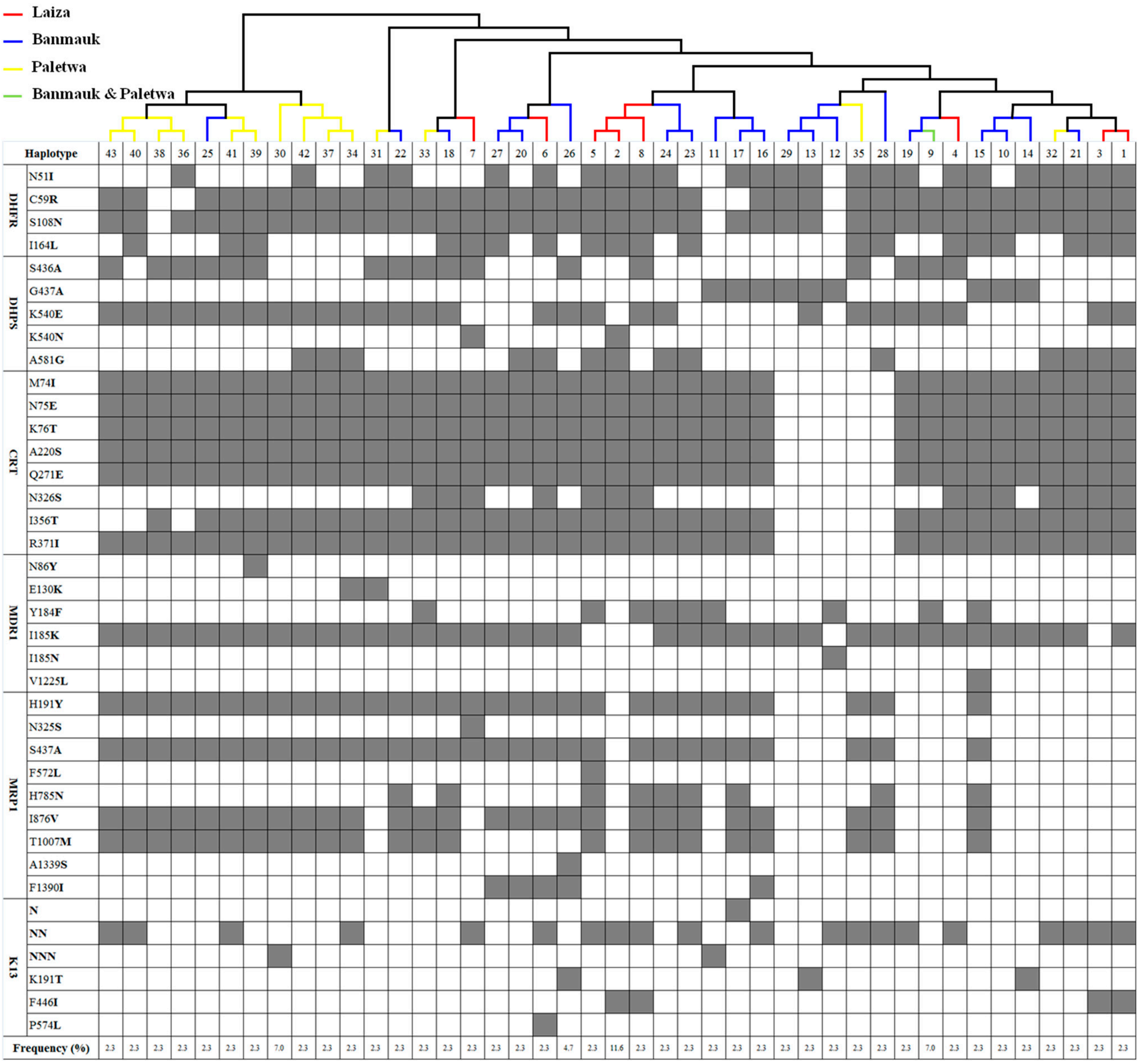

Figure S3. Phylogenetic clustering of the haplotypes based on the mutations in six genes associated with drug resistance of the *P. falciparum* isolates from asymptomatic carriers. A total of 43 haplotypes were identified. Parasites were color-coded by the townships of collection. Haplotypes are shown with mutations in each gene highlighted in grey-filled blocks and wild-type residues shown as empty blocks. Frequencies (%) of haplotypes are shown in the bottom row. *The order of haplotypes is generated automatically through DnaSP.

References

1. Ashley, E.A.; Dhorda, M.; Fairhurst, R.M.; Amaratunga, C.; Lim, P.; Suon, S.; Sreng, S.; Anderson, J.M.; Mao, S.; Sam, B., et al. Spread of artemisinin resistance in Plasmodium falciparum malaria. *The New England journal of medicine* **2014**, *371*, 411-423, doi:10.1056/NEJMoa1314981.
2. Chaijaroenkul, W.; Ward, S.A.; Mungthin, M.; Johnson, D.; Owen, A.; Bray, P.G.; Na-Bangchang, K. Sequence and gene expression of chloroquine resistance transporter (pfcrt) in the association of in vitro drugs resistance of Plasmodium falciparum. *Malaria journal* **2011**, *10*, 42, doi:10.1186/1475-2875-10-42.
3. Das, S.; Mahapatra, S.K.; Tripathy, S.; Chattopadhyay, S.; Dash, S.K.; Mandal, D.; Das, B.; Hati, A.K.; Roy, S. Double mutation in the pfmdr1 gene is associated with emergence of chloroquine-resistant Plasmodium falciparum malaria in Eastern India. *Antimicrobial agents and chemotherapy* **2014**, *58*, 5909-5915, doi:10.1128/AAC.02762-14.
4. Yang, Z.; Li, C.; Miao, M.; Zhang, Z.; Sun, X.; Meng, H.; Li, J.; Fan, Q.; Cui, L. Multidrug-resistant genotypes of Plasmodium falciparum, Myanmar. *Emerging infectious diseases* **2011**, *17*, 498-501, doi:10.3201/eid1703.100870.
5. Dahlstrom, S.; Ferreira, P.E.; Veiga, M.I.; Sedighi, N.; Wiklund, L.; Martensson, A.; Farnert, A.; Sisowath, C.; Osorio, L.; Darban, H., et al. Plasmodium falciparum multidrug resistance protein 1 and artemisinin-based combination therapy in Africa. *The Journal of infectious diseases* **2009**, *200*, 1456-1464, doi:10.1086/606009.
